# A surgical strategy for intrahepatic cholangiocarcinoma — the hilar first concept

**DOI:** 10.1007/s00423-023-03023-y

**Published:** 2023-08-07

**Authors:** Nora Nevermann, Julia Bode, Maxine Vischer, Lina Feldbrügge, Sebastian Knitter, Felix Krenzien, Uwe Pelzer, Uli Fehrenbach, Timo Alexander Auer, Georg Lurje, Moritz Schmelzle, Johann Pratschke, Wenzel Schöning

**Affiliations:** 1 Department of Surgery, Campus Charité-Mitte and Campus Virchow-Klinikum, Charité – Universitätsmedizin Berlin, corporate member of Freie Universität Berlin, Humboldt-Universität Zu Berlin, and Berlin Institute of Health, Augustenburger Platz 1, 13353 Berlin, Germany; 2 Clinical Scientist Program, Berlin Institute of Health (BIH), Anna-Louisa-Karsch-Str. 2, 10178 Berlin, Germany; 3 Department of Hematology, Oncology and Tumorimmunology, Charite Universitatsmedizin Berlin, Berlin, Germany; 4 Department of Radiology, Charité - Universitätsmedizin Berlin, Berlin, Germany

**Keywords:** Intrahepatic cholangiocarcinoma, Liver surgery, Oncologic surgery, Multimodal treatment

## Abstract

**Purpose:**

The present study assesses long-term overall survival (OS) and disease-free survival (DFS) after curative resection for intrahepatic cholangiocarcinoma (ICCA) depending on resection margin (RM) status and lymph node (LN) status.

**Methods:**

Clinical data of all consecutively resected patients with ICCA at a single high-volume center between 2005 and 2018 were collected. Minimum follow-up was 36 months. Perioperative and long-term oncological outcome was assessed.

**Results:**

One hundred ninety-two cases were included in the analysis. Thirty- and 90-day-mortality was 5.2% (*n* = 10) and 10.9% (*n* = 21). OS was 26 months with 1-, 2-, and 5-year-OS rates of 72%, 53%, and 26%. One-, 2-, and 5-year-DFS rates were 54%, 42%, and 35% (N0 vs. N1: 29 vs. 9 months, *p* = 0.116). R1 was not found to be an independent risk factor for reduced survival in the overall cohort (*p* = 0.098). When differentiating according to the LN status, clear resection margins were significantly associated with increased DFS for N0 cases (50 months vs. 9 months, *p* = 0.004). For N1 cases, no significant difference in DFS was calculated for R0 compared to R1 cases (9 months vs. 9 months, *p* = 0.88). For N0 cases, clear resection margins > 10 mm were associated with prolonged OS (*p* = 0.048).

**Conclusion:**

For N1 cases, there was no significant survival benefit when comparing R0 versus R1, while the complication rate remained high for the extended resection types. In view of merging multimodal treatment, the hilar first concept assesses locoregional LN status for optimal surgical therapy.

**Supplementary Information:**

The online version contains supplementary material available at 10.1007/s00423-023-03023-y.

## Introduction

Unanimous consensus exists that surgical resection represents the only potentially curative treatment option for intrahepatic cholangiocarcinoma (ICCA). Nevertheless, a debate remains on the risk–benefit-calibration between extended surgery aiming for clear resection margins (R0) and a less aggressive approach associated with a lower perioperative morbidity and mortality. This debate gains further importance as treatment of ICCA is moving away from a monomodal surgical strategy and towards a multimodal oncological pathway. Adjuvant systemic therapy is nowadays recommended after curative resection [1, 2], and numerous clinical trials have been registered that evaluate the use of targeted therapies (e.g., NCT04361331, NCT03230318, NCT04301778, NCT04989218). Postoperative complications lead to a prolonged length of hospital stay and hinder or delay adjuvant treatment. The present study addresses these conflicting priorities and aims to contribute to an updated surgical strategy.

The impact of microscopically clear resection margins on long-term outcome remains unclear. Several studies describe a significant improvement of disease free survival (DFS) and overall survival (OS) associated with R0 [3–5], while other studies report contrary results [6, 7]. Furthermore, a potential role of the width of clear resection margins remains debated. Tumor free margins of > 10 mm have been described as an independent factor of OS [5, 6, 8], while other studies fail to show a significant association between margin width and long-term survival. [4]

These conflicting results might be caused by interference of other negative prognostic factors. Positive LN status has been described as the most relevant negative prognostic marker for survival (hazard ratio 2.1). [9] For perihilar cholangiocarcinoma, our study group has recently shown a missing benefit for R0 resections for N + cases [10]. First evidence is available for according results for intrahepatic cholangiocarcinoma. [6] Available data is however scarce.

Our study offers an in-detail analysis of long-term OS and DFS depending on RM status, width of resection margins, and LN status. We hereby aim to contribute to an optimized surgical strategy for the specific entity of ICCA based on parameters that can be assessed pre- or intraoperatively.

## Methods

### Study design

Clinical data of all consecutive cases of partial liver resection for ICCA that were performed at the Department of Surgery, Campus Charité-Mitte and Campus Virchow-Klinikum, Charité Universitätsmedizin Berlin, between January 1st of 2005 and December 31st of 2018 was collected in a retrospective design. Inclusion criteria were defined as patients older than 18 years, resectable ICCA without distant metastases, presence of three or more lymph nodes in the histopathological examination, and availability of all relevant clinical data as listed below. Minimum follow-up was defined at 36 months. Patients that underwent surgery for recurrence of ICCA and non-curative resections were excluded from our study.

Ethical approval of the Charité’s Ethics Committee was obtained (EA2/238/20), and all data was collected, stored, and processed according to the local data protection laws. The study was conducted in accordance with the ethical standards of the Helsinki Declaration (1975).

### Data collection and parameters of outcome

Baseline characteristics and data on patients’ physical status and the underlying pathology were collected as well as parameters of intraoperative and postoperative outcome and oncological follow-up. Baseline characteristics included age, sex, body mass index (BMI), the Charlson Index of comorbidities, and the American Society of Anesthesiologists’ Physical Status Classification (ASA score). The presence of liver fibrosis was defined as part of the histopathological examination and graded according to the classification of Desmet and Scheuer. Cirrhosis was defined as a fibrosis grade IV. The Child–Pugh score was used to assess the severity of liver cirrhosis. Tumor size was defined as the diameter of the largest tumor. Preoperative imagery was assessed, and the cases were categorized morphologically as previously published into mass-forming (MF), periductal-infiltrating (PI), and intraductal growing (IG) ICCA [11]. Furthermore, the clinical LN status (cN) was determined. Data on postoperative complications were collected and graded according to the Clavien-Dindo classification. Major complications were defined as Clavien-Dindo ≥ IIIa. Postoperative bile leak was graded according to the International Study Group of Liver Surgery (ISGLS).

Major liver resections were defined as resection of three or more adjacent segments [12]. Procedures were grouped according to the extent of the parenchymal resection into the categories trisectionectomy, right and left hepatectomy, sectionectomy, and minor resections.

Furthermore, RM status was further grouped into three categories of positive resection margins (R1), clear resection margins < 10 mm, and clear resection margins ≥ 10 mm.

Thirty- and 90-day mortality, as well as the length of stay (LOS), length of intensive care unit stay (ICU-LOS) and complications rates were recorded as parameters of perioperative outcome. Overall and disease-free survivals were assessed as parameters of long-term outcome depending on the influence of resection margin and lymph node yield.

### Perioperative management

Perioperative management was individualized but routinely included preoperative computed tomography and/or magnetic resonance imaging of the chest and abdomen. The surgical indication as well as the indication for adjuvant treatment was confirmed by our institutions multidisciplinary tumor board. Patients that received major liver surgery were routinely admitted to ICU for postoperative surveillance. Abdominal drains were routinely placed in close proximity with the parenchymal resection plane and biliary or vascular reconstructions, if applicable. Without pathological findings, drains were removed 48 h after surgery.

Regimes for adjuvant chemotherapy included monotherapy with capecitabine, combination therapy with gemcitabine and cisplatin or since 2014 inclusion in the ACTICCA study (NCT02170090). Our actual institutional standard is — depending on the postoperative patients’ fitness — monotherapy with capecitabine (along the lines of the BILCAP-study results [13]) or screening for the ACTICCA trial.

### Statistical analysis

Counts and proportions were used to report categorical variables and median and interquartile range (IQR) for continuous variables. The Wilcoxon rank-sum test was used to analyze continuous variables and Pearson chi square test for categorical variables. Univariate and multivariate Cox regression analysis was performed to assess the parameters’ impact on outcome. Kaplan–Meier analysis was performed and compared with the log-rank test. Cases of surgery related death were considered as censored for oncological outcome analyses. A *p* value < 0.05 was considered statistically significant. IBM SPSS Statistics version 25 (IBM Corp., Armonk, NY, USA) was used for data analysis.

## Results

### Study population

One hundred ninety-two cases met the inclusion criteria and were included in the analysis.

Median age at surgery was 64 years, and the mean follow-up was 40 months (KI95 33–46 months). For 56% of patients, previous abdominal surgery was reported. Twenty-seven cases (14%) received hypertrophy inducing interventions prior to surgery with either portal vein embolization (*n* = 23) or associating liver partition with portal vein ligation for staged hepatectomy (ALLPS, *n* = 4). Eleven cases (5.7%) received neoadjuvant systemic therapy. Laparoscopic surgery was performed in 3% of all cases. One hundred seventy-seven cases (92%) required major liver resection with trisectionectomy (31%, *n* = 59), left hepatectomy (31%, *n* = 60), and right hepatectomy (30%, *n* = 58) representing the most frequent types of resections. Thirty-two patients (17%) received adjuvant chemotherapy. Baseline characteristics are listed in supp. table 3.

### Preoperative morphologic diagnostics

Preoperative imagery data was available for 171 cases (89%). Median delay between diagnostics and surgery was 3.2 weeks. Morphologic subcategorization showed 83% (*n* = 141) MF, 2% (*n* = 4) PI, and 15% (*n* = 26) IG ICCAs. cN + was found in 33% (*n* = 57). Forty-nine percent (*n* = 37) of pN + were detected by imagery and categorized as cN + , while 51% (*n* = 39) were falsely negative in preoperative imaging. cN was falsely positive in 35% (*n* = 20) of cases with N + not confirmed by histology.

### Perioperative outcome

**Fig. 1:**
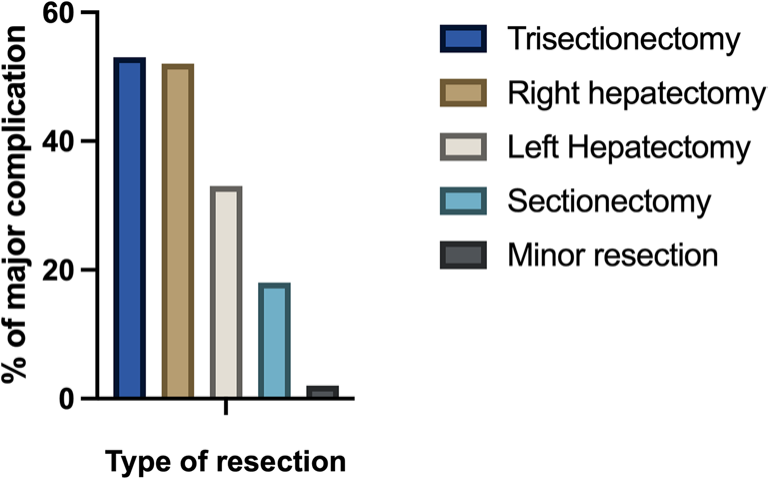
Rate of major complications (Clavien-Dindo ≥ IIIa) depending on type of resection

Thirty- and 90-day-mortality was 5.2% (*n* = 10) and 10.9% (*n* = 21) with median length of hospital stay at 18 days (IRQ: 16 days) and median length of intensive care unit (ICU) stay being 2 days (IQR: 3 days). A major complication defined as Clavien-Dindo ≥ IIIa occurred in 45% (*n* = 87), bile leak representing the most frequent complication (27%, *n* = 52). A significant correlation was found between the extent of parenchymal resection and the rate of major complications (*p* = 0.017, Fig. 1).

A hepaticojejunostomy was performed in 32% (*n* = 61) with a resulting morbidity of 57% (major complications) and a 90-day mortality of 15%. Cases that required combined reconstruction of a major vessel and the bile duct (*n* = 16) showed a rate of major complication of 63% (*p* = 0.069 compared to no reconstruction).

### Oncological outcome and rate of recurrence

Clear margin resection (R0) was achieved in 128 cases (66.7%). The absence of lymph node metastases (N0) was confirmed in 106 cases (55.2%). Median number of harvested LN was 7 (IQR: 5). No association was found between tumor size and prevalence of lymph nodes metastases (*p* = 0.453). Median tumor diameter was 78 mm for N0 cases compared to 83 mm for N1 cases (*p* = 0.461).

Median overall survival was 26 months with 1-, 2-, and 5-year-OS rates at 72%, 53%, and 26%. For N0 versus N1 cases, median OS was 42 versus 17 months (*p* < 0.0001).

In 103 cases (54%), recurrence was recorded with a median DFS of 15 months. 1-, 2-, and 5-year-DFS rates were 54%, 42%, and 35%. For N0 versus N1 cases, median DFS was 29 vs. 9 months (*p* = 0.116).

### Tumor-free resection margins in the context of lymph status

Univariate regression analysis revealed significantly reduced DFS and OS for the following parameters:

- LN status (*p* = 0.002 and 0.001),
- T3 or T4 status (*p* < 0.000 each),
- Lymphovascular (*p* = 0.01 and 0.014) and
- Microvascular invasion (*p* < 0.000).

R1 was not found to be an independent parameter for reduced survival in the overall cohort (OS: HR 1.13, *p* = 0.098; DFS: HR 1.498, *p* = 0.052). When differentiating according to the LN status, a HR of 2.2 was calculated for reduced DFS for N0 R1 cases compared to N0 R0 cases (*p* = 0.012) with an according trend for OS (HR: 1.5, *p* = 0.191).

In N1 cases, R1 was no negative prognostic marker for DFS or OS (*p* = 0.879 and 0.354). Supplementary table 1 and 2 depict the results of uni- and multivariate Cox regression analyses for OS and DFS.

**Fig. 2:**
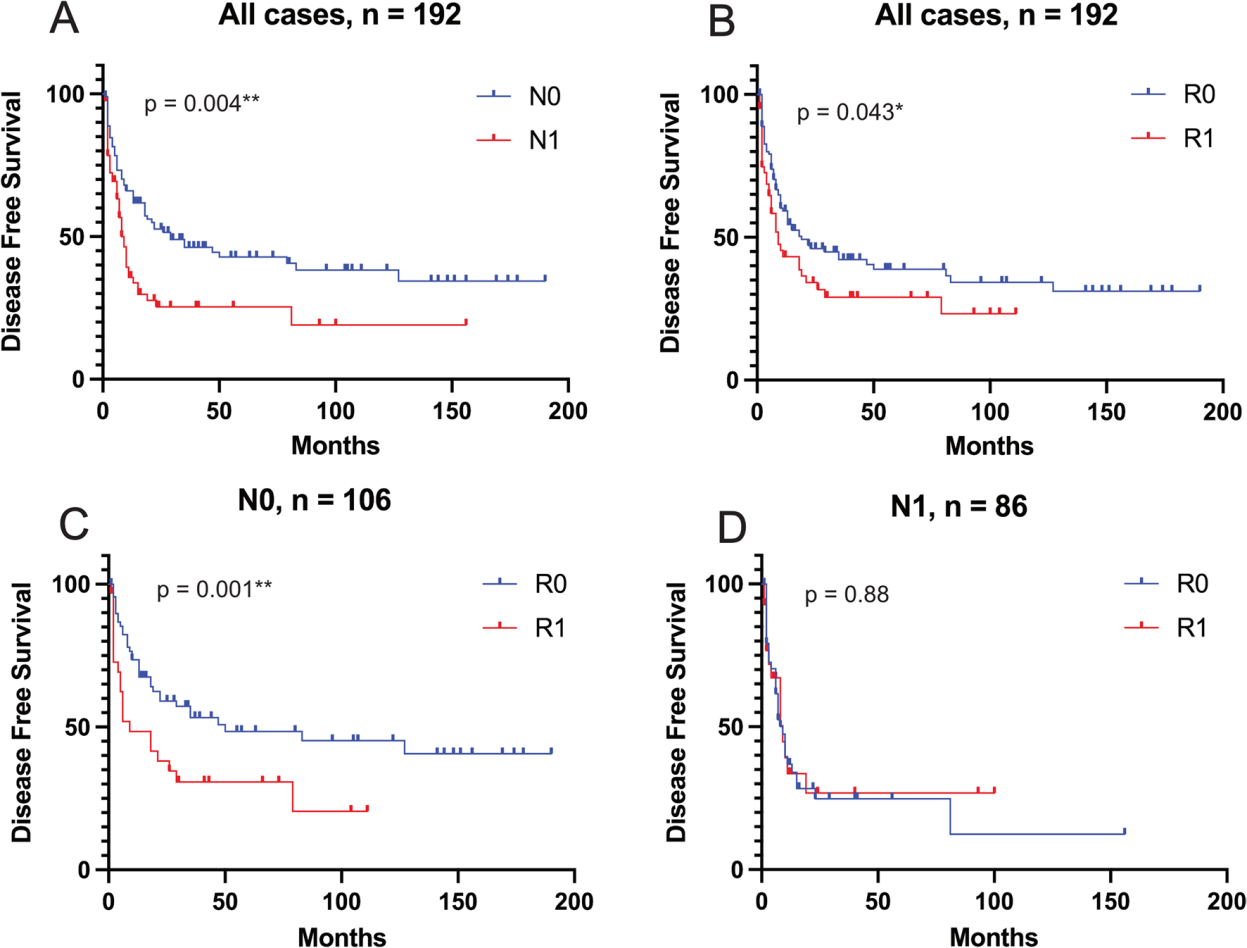
Kaplan–Meier-plots for disease-free survival. **A** Comparison of negative (N0) vs. positive (N1) lymph node status, all cases. **B** Comparison of clear (R0) vs. microscopically invaded (R1) resection margin, all cases. **C** R0 vs. R1 for N0 cases. **D** R0 vs. R1 for N1 cases

**Fig. 3:**
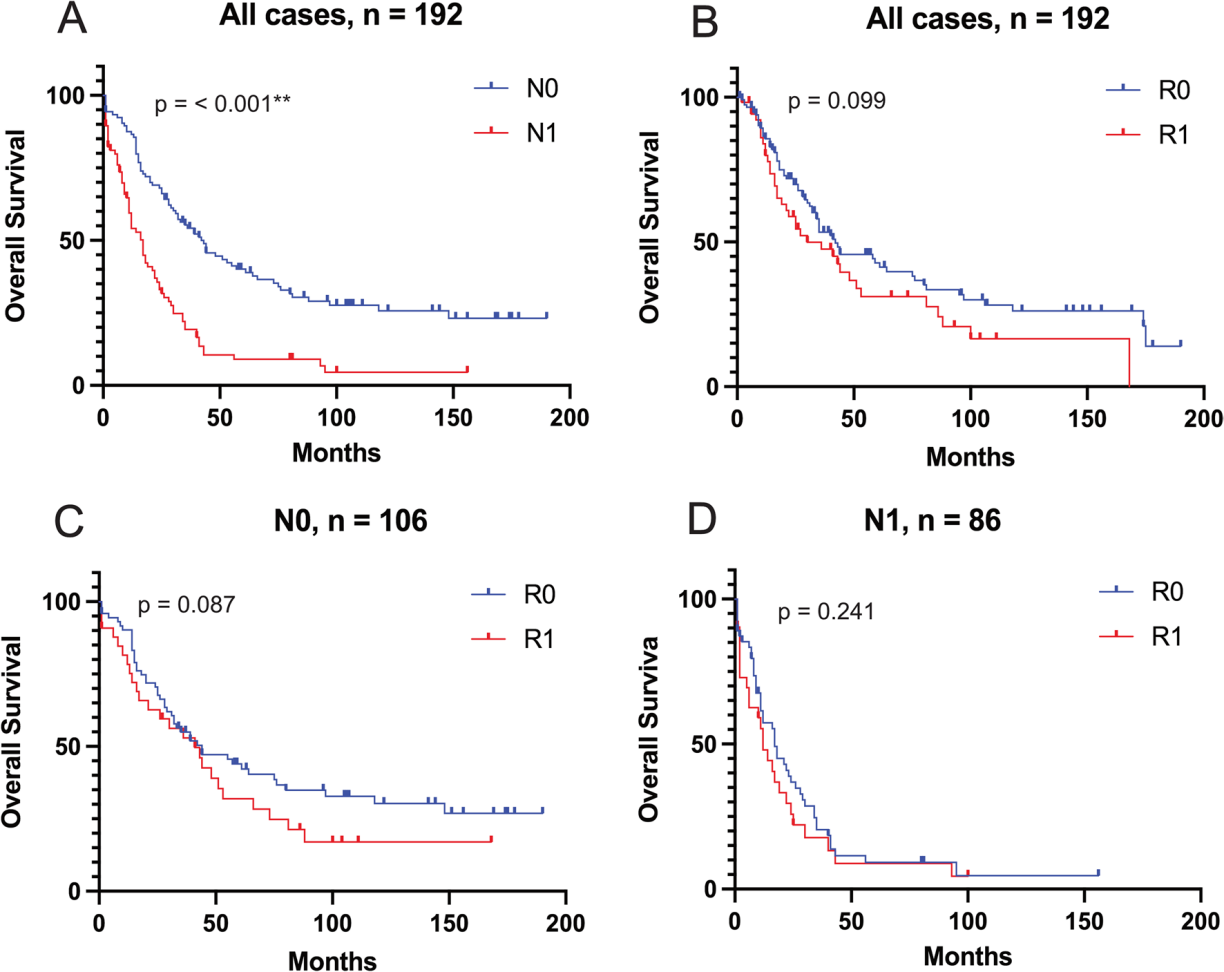
Kaplan–Meier-plots for overall survival. **A** Comparison of negative (N0) vs. positive (N1) lymph node status, all cases. **B** Comparison of clear (R0) vs. microscopically invaded (R1) resection margin, all cases. **C** R0 vs. R1 for N0 cases. **D** R0 vs. R1 for N1 cases

In a next step, Kaplan–Meier analyses were performed to assess survival rates depending on resection margins and LN status. Within the N0 group, clear resection margins were significantly associated with increased DFS (50 months vs. 9 months, *p* = 0.004). For N1 cases, no significant difference in DFS was found for R0 compared to R1 cases (9 months vs. 9 months, *p* = 0.88), Fig. 2. Median OS for N0 with R0 versus R1 was 44 versus 41 months (*p* = 0.087) compared to 17 versus 12 months for N1 cases (*p* = 0.241), Fig. 3.

**Fig. 4:**
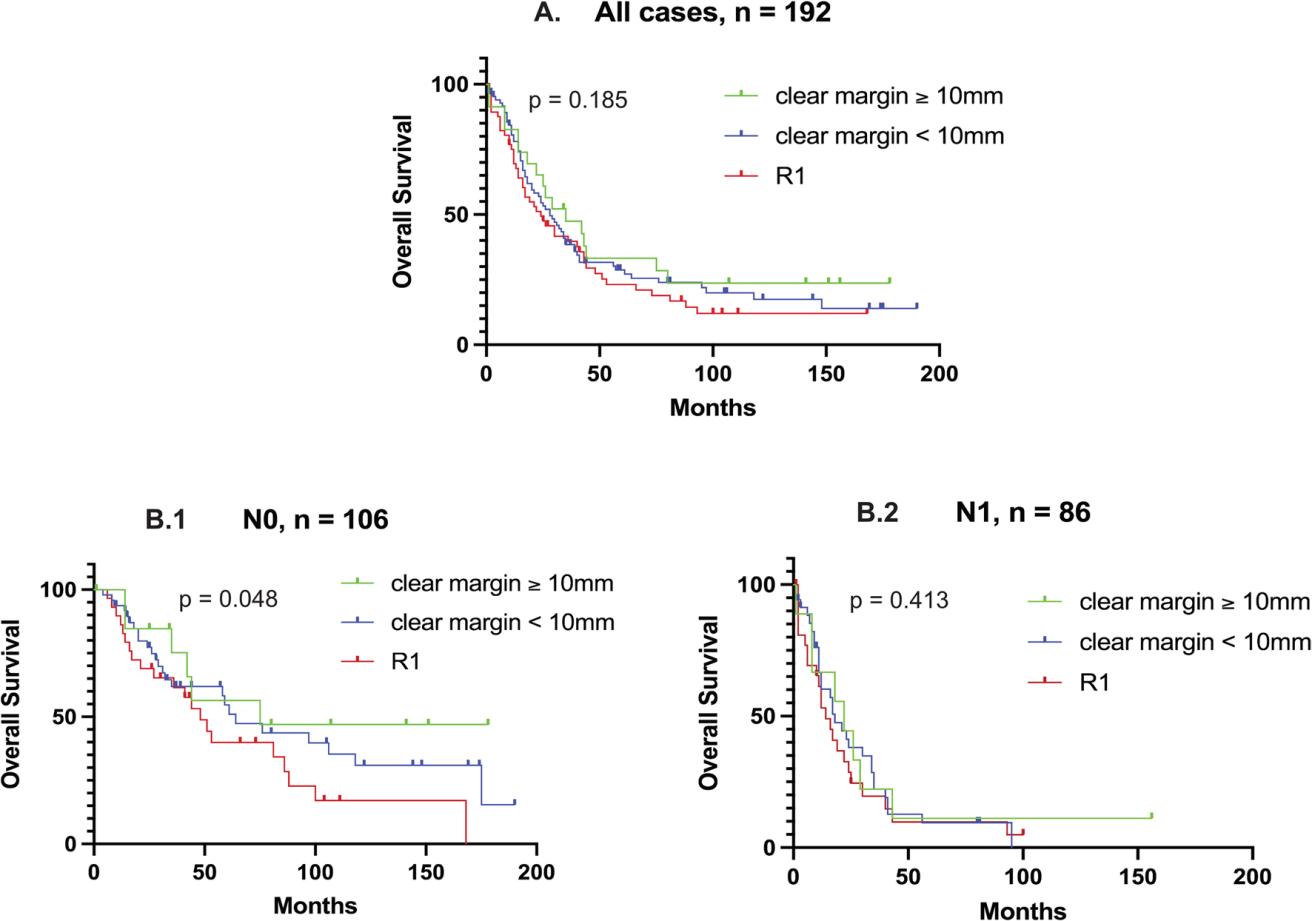
Comparison of Kaplan–Meier-plots for overall survival depending on margin categories and lymph node status

Furthermore, we examined the role of width of microscopically free resection margins depending on LN status. As described above, margin categories were defined as group 1 (free resection margin ≥ 10 mm), group 2 (free resection margin < 10 mm), and group 3 (R1). For the margin groups 1–3, median OS was 35 months, 28 months, and 24 months (*p* = 0.185). For the N0 group, the increase of OS with wider clear resection margins became significant with 75 months, 64 months, and 48 months for margin groups 1–3, *p* = 0.048. For N1 cases, no significant increase was observed, Fig. 4.

Accordingly, a significantly increased DFS was found for N0 cases with increasing width of the resection margin (clear margins ≥ 10 mm: 50 months, clear margins < 10 mm: 47 months, R1: 9 months, *p* = 0.026). For N1 cases, no significant increase of DFS was found for the three margin categories with median DFS of 9 months, 7 months, and 8 months (*p* = 0.872).

### Preoperative imaging and prognostics

In univariant regression, cN + was found to be associated with a shorter OS survival (OR = 2.4, *p* = 0.032). Association with reduced DFS was found to be non-significant (OR = 1.07, *p* = 0.248). Median OS for cN + (*n* = 57) vs. cN- (*n* = 114) was 19 vs. 35 months, *p* = 0.014. Median DFS was 10 vs. 19 months (*p* = 0.201).

**Fig. 5:**
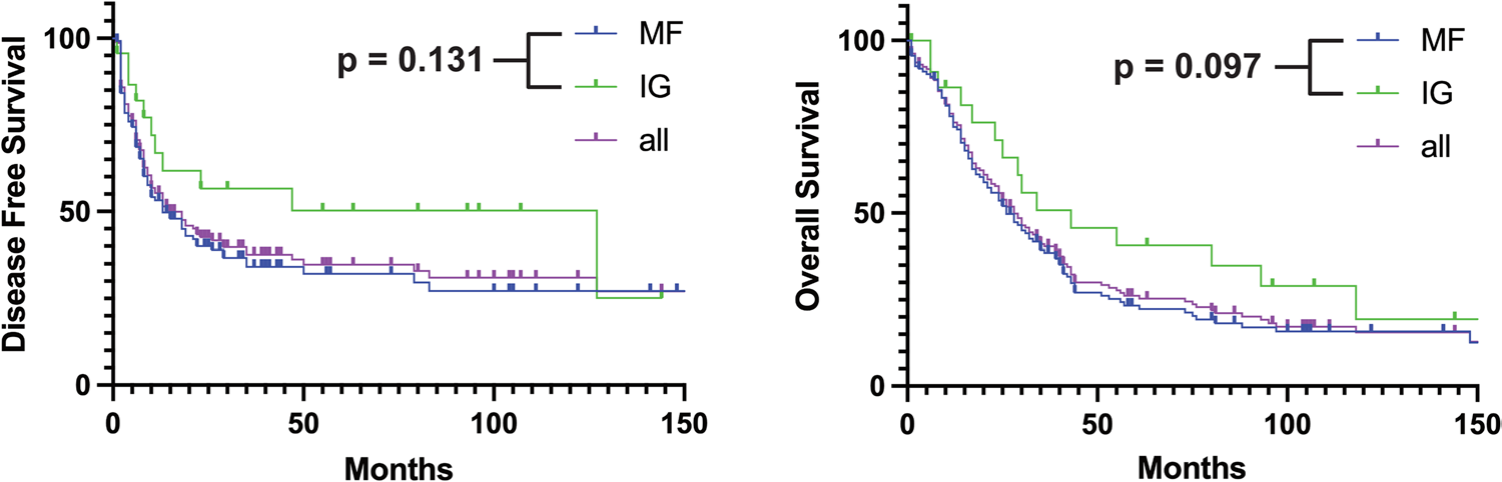
Comparison of Kaplan–Meier-plots for overall survival and disease-free survival depending on morphologic subtype assessed in preoperative imaging (CT/MRI)

When differentiating according to morphological subtype, mass-forming vs. intraductal-growing ICCAs showed a median OS of 26 vs. 43 months, *p* = 0.131, and a median DFS of 13 vs. 127 months, *p* = 0.097. Kaplan–Meier analyses are shown in Fig. 5. Due to a low case number (*n* = 4), periductal-infiltrating ICCAs were excluded from analyses.

### Adjuvant chemotherapy

**Fig. 6:**
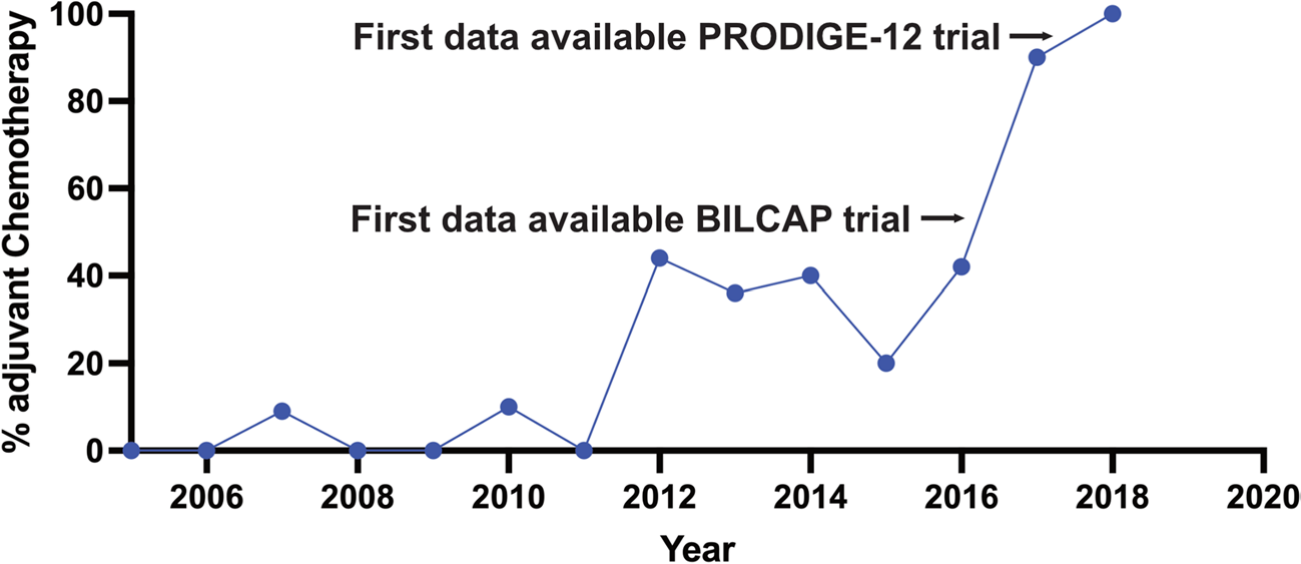
Timeline of fractions that received adjuvant chemotherapy. Publication of the relevant BILCAP [14] and PRODIGE-12 [15] trials are marked

In a next step, the impact of adjuvant chemotherapy depending on LN and RM status was evaluated. One hundred twenty-seven cases could be included in the analysis of which thirty-two patients received adjuvant chemotherapy. Sixty-five cases (34%) had to be excluded due to missing data. Gemcitabine/cisplatin regimen was administered in 19 cases (59%), two patients (6%) were included in the ACTICCA trial, and 11 cases (34%) received capecitabine mono. We observed a significant increase of carried out adjuvant chemotherapy for patients treated before versus after 2017, the time point of the BILCAP trial study completion (13.5% versus 78.3%, *p* < 0.000) [14]. A further increase was observed after 2017 (completion of PRODIGE-12 trial [15]) with 91% of patients receiving adjuvant treatment (*p* < 0.000, s. Figure 6).

No significant difference was found for the baseline characteristics age, sex, BMI, and TNM-classification including V, L, Pn status, and histological grading (s. supplementary table 3) between the cohort that received adjuvant chemotherapy (chemo +) and the cohort without adjuvant chemotherapy (chemo −).

**Fig. 7:**
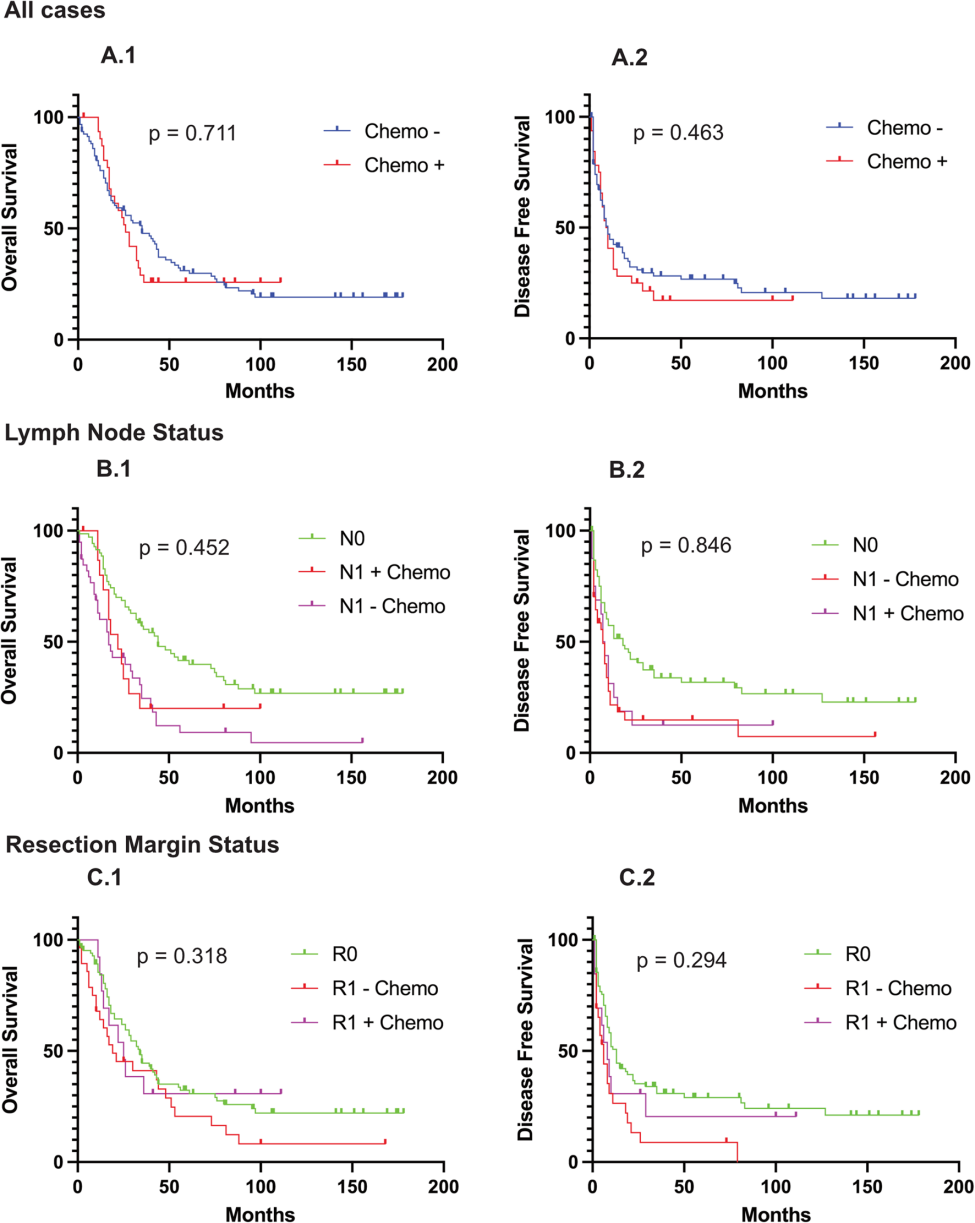
Kaplan–Meier plots for OS and DFS depending on adjuvant treatment, lymph node status and resection margins. **A** OS (A.1) and DFS (A.2) for all cases differentiated according to received (Chemo +) and not received (Chemo −) adjuvant treatment. **B** Impact of adjuvant chemotherapy depending on OS (B.1) and DFS (B.2) for cases with N + compared to N0. **C** Impact of adjuvant chemotherapy depending on OS (C.1) and DFS (C.2) for cases with R1 compared to R0

No significant difference in OS and DFS was found comparing the two cohorts, *p* = 0.711 and 0.463. For N1 cases, a trend for outcome improvement after adjuvant chemotherapy was calculated (OS (chemo − vs. chemo +): 17 vs. 22 months, *p* = 0.452; DFS: 7 vs. 8 months, *p* = 0.846), but the values failed to reach statistical significance. According results were found for the subgroup of R1 cases with OS at 19 versus 25 months (*p* = 0.318) and DFS at 6 versus 8 months (*p* = 0.294). Kaplan–Meier analyses are shown in Fig. 7.

## Discussion

Our study offers a comprehensive analysis of perioperative and oncological outcome depending on lymph node and resection margin status.

The results suggest that an individualized strategy might be appropriate for defining the extent and radicality of surgery. The LN status represents an important prognostic marker for ICCAs as shown in the present study and supported by available data [6, 8]. More aggressive surgery — including resection of a major vessel or bile duct — might entail a relevant survival benefit for N0 cases. Whereas N1 cases might benefit from a less extensive surgery and associated lower perioperative morbidity.

In the context of merging systemic treatment options including immunotherapy [16], a lower risk tolerance for perioperative morbidity has to be discussed especially for cases with an oncological high-risk profile. The benefit of a rapid transition to additive systemic treatment might emerge as a relevant factor. For a custom-tailored surgical concept, we propose the hilar-first concept including based on intraoperative evaluation of the LN status.

The incidence of lymph node metastases in resectable ICCA is described at 30–40% [4, 17] and was 50% in the present study. Our results suggest that preoperative assessment of clinical LN status does not offer sufficient diagnostic power to determine LN status (sensitivity: 49%, specificity: 79%). These findings are in accordance with previously published data [18]. Also other parameters such as tumor size or morphologic subclassification do not contribute significantly to the clinical lymph node assessment.

**Fig. 8:**
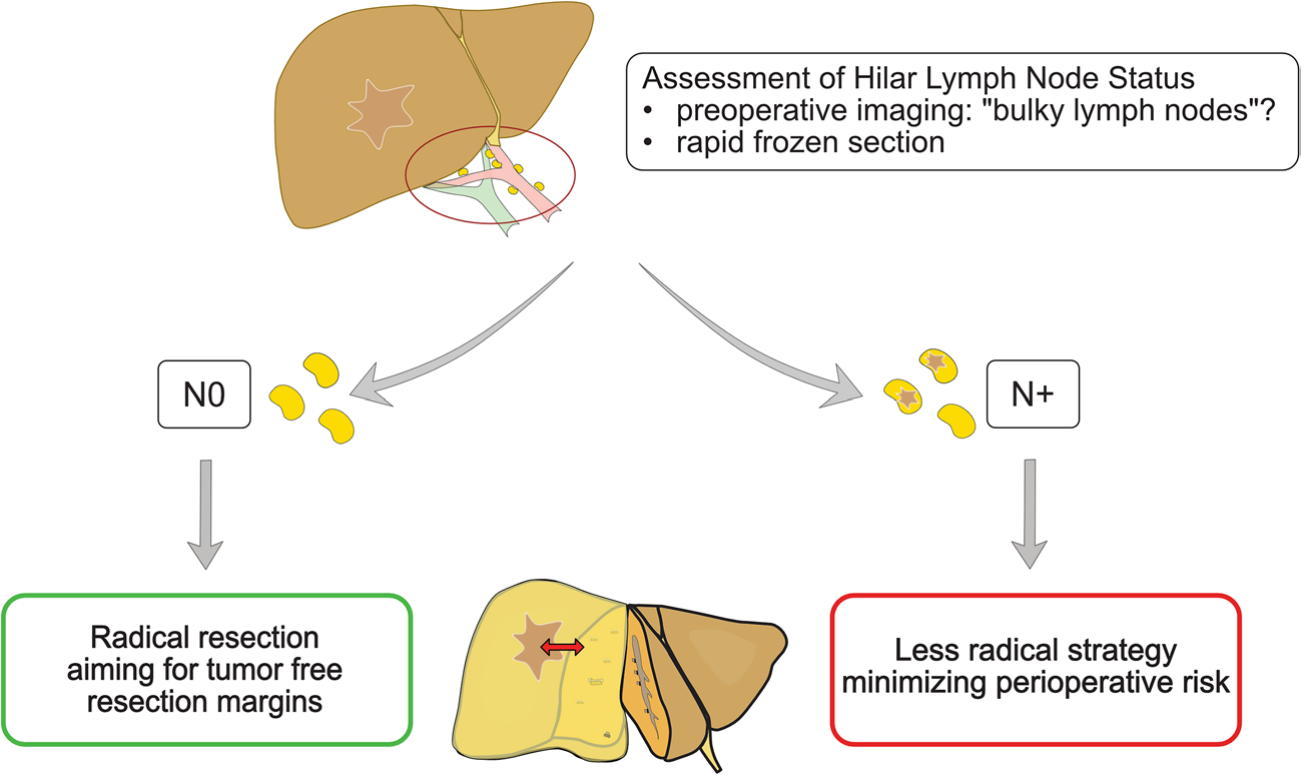
Proposed algorithm for individual surgical strategies based on lymph node status

Given the insufficient diagnostic power of morphologic assessment, we propose an algorithm that includes intraoperative LN status evaluation as visualized in Fig. 8.

In this algorithm, one would start surgery with minimal-invasive radical lymphadenectomy and frozen section analysis of the retrieved lymph nodes. In case of N + , the planed resection might be changed to a less radical procedure (e.g., a smaller FRM and preservation of a greater liver remnant) to facilitate postoperative recovery and enable earlier and more aggressive systemic therapy. In case of N0, the benefit of wider resection margins (> 1 cm) might justify a more radical procedures or even re-resections during the same operation in the case of lesser margins after intra-operative frozen section of the liver specimen.

Our study recorded a major postoperative complication for more than a third of cases that potentially delays the initiation of systemic therapy. Our results lay hereby in a realistic spectrum. According to a recently published text book outcome (TO) for the entity (achievement expected in 9.3–53%) [19], TO was achieved in 40 cases (21%) of our cohort. R0 at was given in 82%, 30-day mortality was calculated at 4.4% and rate of complications at 40%. These numbers emphasize the importance of prudent risk–benefit calculation.

First promising results from trials with targeted therapies in case of specific tumorgenetic alterations (e.g., FGFR2 fusions or IDH-2 alterations) [20, 21] for the unresectable setting should encourage us to investigate these modern substances in a neoadjuvant setting in case of borderline resectability and oncological high-risk profile. Initial genomic tumor profiling from resection specimens might enable a rapid start of targeted therapy in case of disease recurrence.

The ESMO guideline (2016) pronounced a first recommendation for adjuvant treatment for ICCA [22]. Since then, the BILCAP and PRODIGE-12 trials offered data on cytostatic adjuvant treatment for ICCA. We observe a significant increase of administered adjuvant chemotherapy after completion of two hallmark studies reflecting the vast impact of those studies on clinical management. Based on the BILCAP trial, the 2022 clinical guideline by the German Cancer Society on biliary cancer pronounced an indication for adjuvant treatment with capecitabine. No significant increase of survival was found for the subgroup receiving adjuvant chemotherapy, neither for the overall cohort nor after stratification for RM and LN status. Of note, our findings on adjuvant treatment have to be interpreted with caution since less than a fifth of the cohort received adjuvant therapy.

Several limitations have to be discussed. The presented data was collected in a retrospective design. Cases with three or more resected lymph nodes were included according to our inclusion criteria. The number is below the requirement of six or more lymph nodes as defined by the TNM Classification (8th edition). A European multicenter study described a benefit in overall survival after resection of three or more lymph nodes. [23] A second multicenter retrospective study from Korea and Japan confirmed these findings. [24] Based on these data, the chosen inclusion setting allows higher case numbers, but leaves a potential bias for miss-categorizing of the LN status.

Also, our study evaluated the margin towards the parenchymal resection line, but does not consider the possible influence of the tumor’s distance to the liver capsule. Available data do not show a benefit for larger resection margins (> 5 mm) towards the capsule [25], but a resulting bias cannot be excluded.

## Conclusion

Our findings suggest a survival benefit from R0 and clear RM of over 10 mm for N0 cases. In contrast, no significant survival benefit was shown for N1 cases comparing R0 vs. R1. Based on our findings and precedent data, an individualized approach taking into account the intraoperatively assessed LN status might be justified to determine surgical radicalness for ICCAs. Intraoperative frozen section of retrieved lymph nodes might be justified before determination of the resection extent. The increasing role of multimodal treatment emphasizes the importance of a prudent risk–benefit-calculation for extensive surger.

### Supplementary Information

Below is the link to the electronic supplementary material.

Supplementary file1 (DOCX 18 KB)

Supplementary file2 (DOCX 17 KB)

Supplementary file3 (DOCX 17 KB)

## Data Availability

Raw data were generated at Charité Universitätsmedizin Berlin. Derived data supporting the findings of this study are available from the corresponding author on request.

## References

[1] Krenzien F, Nevermann N, Krombholz A, et al (2022) Treatment of Intrahepatic Cholangiocarcinoma-A Multidisciplinary Approach. Cancers (Basel) 14(2) 10.3390/cancers14020362 PMC8773654 35053523

[2] Society) CGoOGC. Diagnostik und Therapie des Hepatozellulären Karzinoms und biliärer Karzi- nome Langversion 3.0, 2022, AWMF-Registernummer: 032/053OL. https://www.leitlinienprogramm-onkologie.de/leitlinien/hcc-und-biliaere-karzinome/. Accessed 15 Sept 2022

[3] Murakami Y, Uemura K, Sudo T (2011). Prognostic factors after surgical resection for intrahepatic, hilar, and distal cholangiocarcinoma. Ann Surg Oncol.

[4] Ribero D, Pinna AD, Guglielmi A (2012). Surgical approach for long-term survival of patients with intrahepatic cholangiocarcinoma: a multi-institutional analysis of 434 patients. Arch Surg.

[5] Spolverato G, Yakoob MY, Kim Y (2015). The impact of surgical margin status on long-term outcome after resection for intrahepatic cholangiocarcinoma. Ann Surg Oncol.

[6] Farges O, Fuks D, Boleslawski E (2011). Influence of surgical margins on outcome in patients with intrahepatic cholangiocarcinoma: a multicenter study by the AFC-IHCC-2009 study group. Ann Surg.

[7] Tamandl D, Herberger B, Gruenberger B, Puhalla H, Klinger M, Gruenberger T (2008). Influence of hepatic resection margin on recurrence and survival in intrahepatic cholangiocarcinoma. Ann Surg Oncol.

[8] Watanabe Y, Matsuyama Y, Izumi N (2020). Effect of surgical margin width after R0 resection for intrahepatic cholangiocarcinoma: a nationwide survey of the Liver Cancer Study Group of Japan. Surgery.

[9] Mavros MN, Economopoulos KP, Alexiou VG, Pawlik TM (2014). Treatment and prognosis for patients with intrahepatic cholangiocarcinoma: systematic review and meta-analysis. JAMA Surg.

[10] Benzing C, Krenzien F, Mieg A (2021). A tailored approach in lymph node-positive perihilar cholangiocarcinoma. Langenbecks Arch Surg.

[11] Bagante F, Spolverato G, Weiss M (2017). Impact of morphological status on long-term outcome among patients undergoing liver surgery for intrahepatic cholangiocarcinoma. Ann Surg Oncol.

[12] Knitter S, Andreou A, Kradolfer D (2020). Minimal-invasive versus open hepatectomy for colorectal liver metastases: bicentric analysis of postoperative outcomes and long-term survival using propensity score matching analysis. J Clin Med.

[13] Primrose JN, Fox RP, Palmer DH (2019). Capecitabine compared with observation in resected biliary tract cancer (BILCAP): a randomised, controlled, multicentre, phase 3 study. Lancet Oncol.

[14] Primrose JN, Fox R, Palmer DH (2017). Adjuvant capecitabine for biliary tract cancer: the BILCAP randomized study. J Clin Oncol.

[15] Edeline J, Benabdelghani M, Bertaut A (2019). Gemcitabine and oxaliplatin chemotherapy or surveillance in resected biliary tract cancer (PRODIGE 12-ACCORD 18-UNICANCER GI): a randomized phase III study. J Clin Oncol.

[16] Park S, Sun JM, Choi YL (2022). Adjuvant durvalumab for esophageal squamous cell carcinoma after neoadjuvant chemoradiotherapy: a placebo-controlled, randomized, double-blind, phase II study. ESMO Open.

[17] de Jong MC, Nathan H, Sotiropoulos GC (2011). Intrahepatic cholangiocarcinoma: an international multi-institutional analysis of prognostic factors and lymph node assessment. J Clin Oncol.

[18] Bartsch F, Hahn F, Muller L (2020). Relevance of suspicious lymph nodes in preoperative imaging for resectability, recurrence and survival of intrahepatic cholangiocarcinoma. BMC Surg.

[19] Merath K, Chen Q, Bagante F (2019). A multi-institutional international analysis of textbook outcomes among patients undergoing curative-intent resection of intrahepatic cholangiocarcinoma. JAMA Surg.

[20] Subbiah V, Iannotti NO, Gutierrez M (2022). FIGHT-101, a first-in-human study of potent and selective FGFR 1–3 inhibitor pemigatinib in pan-cancer patients with FGF/FGFR alterations and advanced malignancies. Ann Oncol.

[21] Abou-Alfa GK, Macarulla T, Javle MM (2020). Ivosidenib in IDH1-mutant, chemotherapy-refractory cholangiocarcinoma (ClarIDHy): a multicentre, randomised, double-blind, placebo-controlled, phase 3 study. Lancet Oncol.

[22] Valle JW, Borbath I, Khan SA (2016). Biliary cancer: ESMO clinical practice guidelines for diagnosis, treatment and follow-up. Ann Oncol.

[23] Sahara K, Tsilimigras DI, Merath K (2019). Therapeutic index associated with lymphadenectomy among patients with intrahepatic cholangiocarcinoma: which patients benefit the most from nodal evaluation?. Ann Surg Oncol.

[24] Kang CM, Suh KS, Yi NJ (2021). Should lymph nodes be retrieved in patients with intrahepatic cholangiocarcinoma? A collaborative Korea-Japan study. Cancers (Basel).

[25] Bartsch F, Baumgart J, Hoppe-Lotichius M, Straub BK, Heinrich S, Lang H (2020). Intrahepatic cholangiocarcinoma - influence of resection margin and tumor distance to the liver capsule on survival. BMC Surg.

