# Supplementary material for: A surgical strategy for intrahepatic cholangiocarcinoma — the hilar first concept
Source: Langenbecks Arch Surg. 2023 Aug 7;408(1):296. doi: 10.1007/s00423-023-03023-y (PMC10404569; doi:10.1007/s00423-023-03023-y)
Supplement: Supplementary file 1 — Supplementary file1 (DOCX 18 KB) [file 423_2023_3023_MOESM1_ESM.docx]

**Supplementary table 2**: Univariate and multivariate regression for all cases, N0 and N1 cases (Overall Survival)

| **All patients, n = 192** | | | | | | |
| --- | --- | --- | --- | --- | --- | --- |
|  | Univariate | | | | Multivariate | |
| Variable | HR (95% CI) | | *P* value | | HR (95% CI) | *P* value |
| T Stage ≥ 3 | 1.823 (1.293 – 2.571) | | 0.001 | | 1.351 (0.938 – 1.945) | 0.106 |
| N Status (N1) | 2.506. (1.788 – 3.511) | | <0.000 | | 2.158 (1.452 - 3.207) | <0.000 |
| Resection margin (R1) | 1.336 (0.948 – 1.882) | | 0.098 | | 1.273 (0.893 – 1.813) | 0.182 |
| Perineural sheath infiltration (Pn1) | 1.485 (0.991 – 2.226) | | 0.055 | | 0.862 (0.546 – 1.362 | 0.525 |
| Lymphovascular invasion (L1) | 1.535. (1.090 – 2.161) | | 0.014 | | 1.073 (0.735 – 1.568) | 0.009 |
| Microvascular invasion (V1) | 2.049 (1.431 – 2.935) | | <0.000 | | 1.669 (1.135 – 2.453) | 0.714 |
| Histopathological Grading |  | |  | |  |  |
| G1 | Reference | |  | |  |  |
| G2 | 1.754 (0.431 – 7.131) | | 0.432 | | * |  |
| G3 | 1.987 (0.479 – 8.247) | | 0.344 | |  |  |
| Adjuvant chemotherapy | 1.566 (0.800 – 3.067) | | 0.191 | | 1.814 (0.880 – 3.741) | 0.107 |
|  |  | |  | |  |  |
| **N0 patients, n = 106** | | | | | | |
|  | Univariate | | | Multivariate | | |
| Variable | HR (95% CI) | *P* value | | HR (95% CI) | | *P* value |
| T Stage ≥ 3 | 1.749 (1.030 – 2.971) | 0.039 | | 1.179 (0.643 – 2.162) | | 0.595 |
| Resection margin (R1) | 1.384. (0.850 – 2.254) | 0.191 | | 1.480 (0.886 – 2.470) | | 0.134 |
| Perineural sheath infiltration (Pn1) | 2.350 (1.192 – 4.634) | 0.014 | | 2.592 (1.228 – 5.468) | | 0.012 |
| Lymphovascular invasion (L1) | 1.035 (0.563 – 1.902) | 0.913 | | 1.063 (0.566 – 1.995) | | 0.850 |
| Microvascular invasion (V1) | 2. 538 (1.448 – 4.449) | 0.001 | | 2.384 (1.275 – 4.455) | | 0.006 |
| Histopathological Grading |  |  | |  | |  |
| G1 | Reference |  | |  | |  |
| G2 | 1.189. (0.288 – 4.914) | 0.811 | | * | |  |
| G3 | 1.235 (0.279 – 5.458) | 0.781 | |  | |  |
| Adjuvant chemotherapy | 2.193 (0.944 – 5.093) | 0.068 | | 2.524 (0.977 – 6.519) | | 0.056 |
|  |  | |  | |  |  |
| **N+ patients, n = 86** | | | | | | |
|  | Univariate | | | Multivariate | | |
| Variable | HR (95% CI) | *P* value | | HR (95% CI) | | *P* value |
| T Stage ≥ 3 | 1.270 (0.795 – 2-030) | 0.318 | | 1.233 (0.750 – 2.029) | | 0.409 |
| Resection margin (R1) | 1.250 (0.771 – 2-026) | 0.365 | | 1.148 (0.676 – 1.947) | | 0.610 |
| Perineural sheath infiltration (Pn1) | 0.769 (0.457 – 1.294) | 0.322 | | 0.650 (0.374 – 1.131) | | 0.610 |
| Lymphovascular invasion (L1) | 1.198 (0.751 – 1.910) | 0.449 | | 1.169 (0.715 – 1.911) | | 0.534 |
| Microvascular invasion (V1) | 1.346 (0.839 – 2.159) | 0.218 | | 1.478 (0.871 – 2.507) | | 0.147 |
| Histopathological Grading |  |  | |  | |  |
| G1 | Reference |  | |  | |  |
| G2 | * |  | | * | |  |
| G3 |  |  | |  | |  |
| Adjuvant chemotherapy | 1.162 (0.385 – 3.501) | 0.790 | | 1.172 (0.372 – 3.695) | | 0.787 |
|  |  |  | |  | |  |
|  |  | |  | |  |  |

*: due to low case numbers for G1 cases, calculation was not possible
