# Supplementary material for: A surgical strategy for intrahepatic cholangiocarcinoma — the hilar first concept
Source: Langenbecks Arch Surg. 2023 Aug 7;408(1):296. doi: 10.1007/s00423-023-03023-y (PMC10404569; doi:10.1007/s00423-023-03023-y)
Supplement: Supplementary file 2 — Supplementary file2 (DOCX 17 KB) [file 423_2023_3023_MOESM2_ESM.docx]

**Supplementary table 3**: Univariate and multivariate regression for all cases, N0 and N1 cases (Disease Free Survival)

| **All patients (N0 and N+)** | | | | | | |
| --- | --- | --- | --- | --- | --- | --- |
|  | Univariate | | | | Multivariate | |
| Variable | HR (95% CI) | | *P* value | | HR (95% CI) | *P* value |
| T Stage ≥ 3 | 2.517. (1.676 – 3.782) | | < 0.000 | | 2.005 (1.282 – 3.137) | 0.002 |
| N Status (N1) | 1.869 (1.257 – 2.778) | | 0.002 | | 1.246 (0.774 – 2.005) | 0.366 |
| Resection margin (R1) | 1.498 (0.997 – 2.251) | | 0.052 | | 1.114 (0.724 – 1.714) | 0.625 |
| Perineural sheath infiltration (Pn1) | 1.891 (1.200 – 2.981) | | 0.006 | | 1.236 (0.739 – 2.069) | 0-420 |
| Lymphovascular invasion (L1) | 1.708 (1.139 – 2.563) | | 0.010 | | 1.356 (0.875 – 2.102) | 0.173 |
| Microvascular invasion (V1) | 2.107 (1.396 – 3.182) | | < 0.000 | | 1.517 (0.985 – 2.336) | 0.059 |
| Histopathological Grading |  | |  | |  |  |
| G1 | Reference | |  | |  |  |
| G2 | 1.754 (0.431 – 7.131) | | 0.432 | | * |  |
| G3 | 1.987 (0.479 – 8.247) | | 0.344 | |  |  |
| Adjuvant chemotherapy | 1.566 (0.800 – 3.067) | | 0.191 | | 1.467 (0.537 – 4.007) | 0.455 |
|  |  | |  | |  |  |
| **N0 patients** | | | | | | |
|  | Univariate | | | Multivariate | | |
| Variable | HR (95% CI) | *P* value | | HR (95% CI) | | *P* value |
| T Stage ≥ 3 | 2.980. (1.664 – 5.337) | < 0.000 | | 2.041 (1.060 – 3.930) | | 0.033 |
| Resection margin (R1) | 2.218 (1.164 – 3.498) | 0.012 | | 1.758 (0.950 – 3.254) | | 0.072 |
| Perineural sheath infiltration (Pn1) | 3.527 (1.688 – 7.371) | 0.001 | | 2.436 (1.028 – 5.776) | | 0.043 |
| Lymphovascular invasion (L1) | 1.252 (0.640 – 2.446) | 0.512 | | 1.310 (0.661 – 2.596) | | 0.439 |
| Microvascular invasion (V1) | 3.070 (1.642 – 5.737) | < 0.000 | | 2.548 (1.310 – 4.959) | | 0.006 |
| Histopathological Grading |  |  | |  | |  |
| G1 | Reference | 0. | | * | |  |
| G2 | 1.189 (0.288 – 4.914) | 0.811 | |  | |  |
| G3 | 1.235 (0.279 – 5.458) | 0.781 | |  | |  |
| Adjuvant chemotherapy | 2.193 (0.944 – 5.093) | 0.068 | | 1.427 (0.334 – 6.096) | | 0.631 |
|  |  |  | |  | |  |
| **N+ patients** | | | | | | |
|  | Univariate | | | Multivariate | | |
| Variable | HR (95% CI) | *P* value | | HR (95% CI) | | *P* value |
| T Stage ≥ 3 | 1.626 (0.911 – 2.902) | 0.100 | | 1.736 (0.935 – 3.225) | | 0.081 |
| Resection margin (R1) | 0.956 (0.519 – 1.763) | 0.886 | | 0.853 (0.441 – 1.650) | | <0. |
| Perineural sheath infiltration (Pn1) | 0.949 (0.519 – 1.737 | 0.866 | | 1.005 (0.533 – 1.895) | | 0.987 |
| Lymphovascular invasion (L1) | 1.473 (0.822 – 2.640) | 0.193 | | 1.321 (0.721 – 2.419) | | 0.368 |
| Microvascular invasion (V1) | 1.244 (0.710 – 2.182) | 0.446 | | 1.119 (0.624 – 2.007) | | 0.706 |
| Histopathological Grading |  |  | |  | |  |
| G1 | * |  | |  | |  |
| G2 |  |  | | * | |  |
| G3 |  |  | |  | |  |
| Adjuvant chemotherapy | 1.162 (0.385 – 3.501) | 0.790 | | 1.205 (0.303 – 4.799) | | 0.791 |
|  |  | |  | |  |  |

*: due to low case numbers for G1 cases, calculation was not possible
