# Supplementary material for: A surgical strategy for intrahepatic cholangiocarcinoma — the hilar first concept
Source: Langenbecks Arch Surg. 2023 Aug 7;408(1):296. doi: 10.1007/s00423-023-03023-y (PMC10404569; doi:10.1007/s00423-023-03023-y)
Supplement: Supplementary file 3 — Supplementary file3 (DOCX 17 KB) [file 423_2023_3023_MOESM3_ESM.docx]

**Supplementary Table 3:** Baseline characteristics

Frequencies are expressed in numbers and percentages, numeric values as medians and interquartile range. Kaplan-Meyer-Analyses were performed for survival assessment. CDK: chronic kidney disease. BMI: body mass index. ASA: ASA (American Society of Anesthesiologists) classification. *cN status based on a case number = 171.

| **BASELINE CHARACTERISTICS** | | **ONCOLOGICAL OUTCOME** | |
| --- | --- | --- | --- |
| **n = 192** | | **n = 192** | |
| Age (years) | 63 (14) | R0 | 128 (67%) |
| Sex (female) | 92 (48%) | N0 | 106 (55%) |
| BMI (kg/m2) | 25.2 (6.6) | Median OS | 26 months |
| Charlson Index | 4 (2) | N0 | 42 months |
| ASA ≥ 3 | 67 (35%) | R0 | 44 months |
| Liver cirrhosis | 11 (6%) | R1 | 41 months |
| CDK | 19 (10%) | N1 | 17 months |
| Previous abdominal surgery | 79 (41%) | R0 | 17 months |
| Diabetes | 36 (19%) | R1 | 12 months |
| Neoadjuvant systemic therapy | 11 (6%) | Median DFS | 15 months |
| Major liver resection | 177 (92%) | N0 | 29 months |
| Trisectionectomy | 59 (31%) | R0 | 50 months |
| Right hepatectomy | 58 (30%) | R1 | 9 months |
| Left hepatectomy | 60 (31%) | N1 | 9 months |
| cN0* | 57/171 (33%) | R0 | 9 months |
| Sensitivity (cN0 pN0) | 49% | R1 | 9 months |
| Specificity (cN1 pN1) | 79% | OS Resection Margins |  |
|  |  | FRM ≥ 10mm | 35 months |
|  |  | N0 | 75 months |
|  |  | FRM < 10mm | 28 months |
|  |  | N0 | 64 months |
| **PERIOPERATIVE OUTCOME** | |  |  |
| **n = 192** | |  |  |
| 30-day-mortality | 10 (5%) |  |  |
| 90-day-mortality | 21 (11%) |  |  |
| Median Hospital Stay | 18 days (16) |  |  |
| Clavien-Dindo ≥ IIIa | 87 (45%) |  |  |
| Bile Leak | 52 (27%) |  |  |
| **SUBGROUP: ADJUVANT CHEMOTHERAPY** | | | |
| **Variable** | **Adjuvant Chemotherapy -** | **Adjuvant Chemotherapy +** | ***p*** |
|  | **n = 95** | **n = 32** |  |
| Age (median, IQR) | 65 (13) | 56 (20) | 0.058 |
| Sex (female) | 50 (52%) | 16 (50%) | 0.478 |
| ASA Scores ≥ 2 | 87 (92%) | 28 (88%) | 0.675 |
| Charlson-Index (median IQR) | 4 (2) | 4 (2) | 0.323 |
| BMI (median IQR) | 25 (7) | 24.8 (9) | 0.744 |
| T Stage ≥ 3 | 31 (33%) | 10 (31%) | 0.506 |
| N Status (N1) | 42% | 50% | 0.012 |
| Resection margin (R1) | 29 (30%) | 13 (41%) | 0.444 |
| Perineural sheath infiltration (Pn1) | 20 (21%) | 5 (16%) | 0.340 |
| Lymphovascular invasion (L1) | 38 (59%) | 9 (41%) | 0.142 |
| Microvascular invasion (V1) | 21 (21%) | 10 (31%) | 0.467 |
| Histopathological Grading |  |  | 0.251 |
| G1 | 2 (3%) | 2 (6%) |  |
| G2 | 58 (62%) | 24 (75%) |  |
| G3 (reference) | 32 (35%) | 6 (19%) |  |
